# Emotional Susceptibility

**Published:** 1868-10

**Authors:** Theo. Griffin

**Affiliations:** 789 State Street


### EMOTIONAL SUSCEPTIBILITY.

PROF. DAVIS, *Dear Sir*: The following case is considered interesting, because it illustrates a degree of nervous susceptibility which individuals rarely possess:—

A few days ago, I was called to see a gentleman who, it was said, had fainted. I found him seated in a chair, supported by a friend; animation entirely suspended; face of cadaveric pallidness; skin bathed in a cold perspiration; extremities cold and pulseless. Had him placed at once in a horizontal position, and applied the usual restoratives. After several minutes, he began to show evidences of returning animation, and soon entirely recovered.

The narration of the details of an operation for the excision of a hemorrhoidal tumor, ungarnished by an attempt to convey an idea of severe suffering, produced this severe shock to the nervous system, with its untoward symptoms.

Can we account for this great disparity between the manifestations of the emotions and the power of the will? This gentleman felt these depressing emotions "swelling up," as he expressed it, and thought to be able to overcome them by an effort of the will. How he succeeded has been shown.

"In a well-balanced condition of the mind, under the ordinary conditions of life, the emotions are controlled by the will," says one. But, are not emotions which are due to the action of the sympathetic system beyond the influence of the will? Doubtless, the leading emotions—grief, joy, etc.—are expressed through this system of nerves, each exercising a peculiar influence upon the circulation: exciting, depressing, or otherwise disturbing it; and are only indirectly under the control of volition. The effect of an emotion cannot be controlled or avoided, except we avoid or control the occurrence of the emotion, by occupying the mind with other thoughts, of a different character, more or less agreeable, as may, at the time, be indicated by the mental condition. A remedy for the cure of anger, which teachers of music prescribe for their pupils, is dictated on this principle: "When you get vexed or angry, *sing.*" And, certainly, if one enters properly into the spirit of a cheerful song, he can entertain no emotion of anger. This is rational therapeutics, undoubtedly.

In the case of the gentleman mentioned, the symptoms observed may be referred to the organic system of nerves acting upon the heart and circulation, producing partial paralysis of the heart's action, resulting in a deficient supply of arterial blood to the great nervous centres, causing fainting, cold extremities, pallor, etc., as a result. He is probably 45 or 50 years of age, and evidently of refined manners and education. How much does education and the manner of life modify emotional, or, if you will, nervous susceptibility?

Truly yours,

THEO. GRIFFIN, M.D., 789 State Street.

